# Maternal and neonatal outcomes after energy-restricted diet for women with gestational diabetes mellitus: A systematic review and meta-analysis of randomized controlled trials

**DOI:** 10.1097/MD.0000000000025279

**Published:** 2021-04-09

**Authors:** Yaofang Feng, Zengcai Zhao, Dayin Fu, Wen Gao, Fei Zhang

**Affiliations:** Department of Obstetrics, Zaozhuang Maternity and Child Health Care Hospital, Zaozhuang, Shandong, P.R. China.

**Keywords:** calorie restriction, dietary intervention, gestational diabetes mellitus, meta-analysis

## Abstract

**Background:**

Gestational diabetes mellitus (GDM) affects 1% to 14% of pregnant women annually worldwide and is one of the most common pregnancy complications.

**Objective:**

We reviewed studies on maternal and neonatal outcomes after dietary managements for women with GDM comparing caloric-restricted (intervention group) and unrestricted diets (control group).

**Methods:**

We systematically searched online databases including Medline, Cochrane Controlled Register of Trials (CENTRAL), ScienceDirect, and Google Scholar from inception until September 2019. We performed a meta-analysis with random-effects model and reported pooled risk ratios (RRs) or pooled mean differences (MD) with 95% confidence intervals (CIs).

**Results:**

We analyzed data from 6 randomized controlled trials including 1300 participants, most of them with high bias risks. We found that the women in the intervention group achieved slightly better glycemic control (pooled MD, −0.72 mg/dL; 95% CI, −7.10 to 5.66 mg/dL) and overall pregnancy outcomes (except neonatal hypoglycemia) than the women in the control group.

**Conclusion:**

An energy-restricted diet does not seem superior to the usual/standard GDM diet based on maternal or neonatal outcomes. But, clinical recommendations cannot be made as the evidence is inconclusive.

## Abstract 2

Supplemental Digital Content is available in the text

## 1 Introduction

Gestational diabetes mellitus (GDM) affects 1% to 14% of pregnant women annually around the globe^[1]^ and is one of the most common pregnancy complications. The prevalence of GDM is high and goes up to 30% in some populations.^[2]^ Resistance to insulin increases with gestational age.^[2]^ Placental hormones such as lactogen, tumor necrosis factor alpha, growth hormones, progesterone, and cortisol are responsible for this insulin resistance.^[3,4]^ Thus, the glucose supply increases in mothers to help fetal growth and development.^[4]^ GDM occurs when the secretion of insulin is inadequate for the degree of resistance.^[3]^

GDM can have a negative impact on both the mother and her fetus/neonate. Adverse outcomes among mothers include preeclampsia, need for labor induction or cesarean section, uterine rupture, cephalopelvic disproportion, perineal lacerations, and shoulder dystocia.^[5–12]^ Mothers with GDM have an approximate risk of developing type 2 diabetes mellitus that is 7 times higher than that of normoglycemic mothers.^[13]^ Adverse outcomes for the fetus/neonate include being large for gestational age or having macrosomia.^[6,9,10,12,14]^ These babies are at higher risk for birth traumas that can lead to perinatal asphyxia, nerve palsies, or bone fractures.^[10,15,16]^ In the long term, these babies also have higher risks of becoming overweight or obese or even developing type 2 diabetes mellitus.^[17,18]^ Other adverse complications include neonatal hypoglycemia, neonatal hyperbilirubinemia, cardiomyopathy, hypomagnesemia, hypocalcemia, and polycythemia.^[10,14,19]^

Many studies show that the GDM outcome for both mothers and new-borns is improved with appropriate metabolic management.^[20]^ During the last decade, this management of women with GDM shifted from endocrinologist-based to diabetes nurse-based care, resulting in substantial reductions in financial burden on the healthcare system and improved glycemic control and postnatal follow-up results.^[21–23]^

Dietary interventions and lifestyle modifications are the primary line of management for mothers with GDM. However, the evidence on the specific nutritional strategies like total energy/caloric intake and distribution of nutrients to manage the GDM is scarce.^[10,24,25]^ Severe caloric restriction and weight loss during pregnancy can increase the risks of ketonemia and development of small for gestational age infants.^[26–29]^ In addition, the degree of restriction of energy/calories for women with pre-pregnancy overweight or obesity for achieving optimal blood glucose control and weight gain during pregnancy is unknown.^[25]^ However, optimal blood glucose levels and weight reduce the listed adverse maternal and fetal/neonatal risks. Ironically, no systematic efforts have been implemented to synthesize the evidence on maternal and neonatal outcomes after different GDM dietary practices. Thus, we designed this meta-analysis to compare the effects of energy-restricted and unrestricted diets on the maternal and neonatal outcomes in mothers with GDM.

## 2 Methods

### 2.1 Type of studies included

We analyzed full texts or abstracts of parallel arm, individual, randomized, quasirandomized, or cluster randomized controlled trials (RCTs), and we excluded unpublished studies or data.

### 2.2 Participants

Pregnant women with GDM.

### 2.3 Type of intervention

We focused on studies comparing the effectiveness of an energy-restricted diet with that of an energy-unrestricted diet.

### 2.4 Types of outcome measure

#### 2.4.1 Maternal outcomes

We calculated glycemic control rates (fasting blood sugar mg/dL), hypertensive disorder of pregnancy rates, modes of delivery (cesarean section), birth traumas, and shoulder dystocia rates in mothers of the 2 groups.

#### 2.4.2 Fetal/neonatal outcomes

We calculated birth weights (in grams), gestational age at delivery (in weeks), rates of large-for-gestational-age babies (neonates with birth weight >90^th^ percentile for gestational age), macrosomia (birth weight >4 kg), perinatal mortality, and neonatal hypoglycemia in new-borns of the two groups.

We selected trials reporting any of those outcomes in control and intervention groups.

#### 2.4.3 Search strategy

We searched the Medline, Google Scholar, ScienceDirect, and Cochrane Central Register of Controlled Trials (CENTRAL), clinical trial registries like ClinicalTrials.gov, and WHO International Clinical Trials Registry Platform (ICTRP) databases using medical subject heading (MeSH) and free text terms to find relevant publications. We used combinations of the following MeSH terms: “Energy Restricted Diet”, “Gestational Diabetes Mellitus”, “Glycaemic Control”, “Neonatal Mortality”, “Pregnancy”, “Dietary Intervention”, and “Randomized Controlled Trial”. Our searches (exclusively in English) ran from inception to September 2019 for all databases.

#### 2.4.4 Additional resources

We checked the reference lists of the database-identified trials for additional relevant articles. We contacted the authors of studies missing data to complete those needed for our assessments.

### 2.5 Data collection and analysis

#### 2.5.1 Study selection

Two investigators independently screened titles, abstracts, and keywords to identify publications meeting the inclusion criteria. Then, the primary and secondary investigators further screened the abstracts and full texts of the retrieved articles independently selecting the studies that satisfied our eligibility criteria for analysis. Any selection process disagreements between the 2 investigators were resolved either through consensus or consultation with a third investigator. The third investigator monitored the overall review process quality. We followed the Preferred Reporting Items for Systematic Review and Meta-Analysis (PRISMA) guidelines to report our review findings.^[30]^

#### 2.5.2 Data extraction and management

The primary investigator collected the necessary study variables for our review recording general information (extraction date, trial title, and authors); methodological details (study type, participants, and setting); participants’ details (sample sizes, baseline and endpoint characteristics, and inclusion and exclusion criteria); intervention variables; follow-up durations; primary and secondary outcomes from each study group with assessment times; and other data for assessing the studies’ quality.

The primary and secondary investigators independently collected the outcome measure data from the studies. The primary investigator transferred the data into the statistical software RevMan (version 5.3), and the third investigator compared the data in the review with those in the trials to ensure their correctness.

#### 2.5.3 Assessment of risk of bias in the studies included

Two independent investigators used the Cochrane risk of bias tool for Randomized Controlled Trials to assess the risk of bias in the publications analyzed.^[25]^ They focused on the following domains for the risk assessment: random sequence generation, allocation concealment, participants’ blinding, incomplete outcome data, outcome assessment blinding, selective outcome reporting, and other risks of bias.

For each of the above-mentioned domains, we assessed the risks of bias as low (adequate data provided), high (inadequate data or not performed), and unclear (missing data).

#### 2.5.4 Statistical analysis

We used RevMan 5.3 (Copenhagen; The Nordic Cochrane Center, The Cochrane Collaboration, 2014) to perform the meta-analysis with the selected studies. For continuous outcomes, we reported the means and standard deviations at follow-up or end line and calculated pooled estimates. Finally, we reported pooled estimates as mean differences with 95% confidence intervals. We calculated the numbers of events and participants in each study arm and entered them into the software and estimated the pooled effect size according to the relative risk (all other outcomes were dichotomous). We applied an inverse variance random-effects model.^[31]^ We contacted the corresponding author of trials with missing data and only followed an imputation method if retrieving the necessary data proved impossible.

#### 2.5.5 Assessment of heterogeneity

We applied a Chi square test of heterogeneity and calculated the *I*^2^ statistic to assess the evidence for between-study variance due to heterogeneity and quantify inconsistencies. We considered an *I*^2^ <25% as mild, one between 25% and 75% as moderate, and one >75% as substantially heterogeneous.^[31]^ We made forest plots to report study-specific and pooled estimates graphically.

#### 2.5.6 Assessment of bias reporting

We checked whether the included trial was registered in a trial registry and whether its full protocol was available, and we compared lists of outcomes in the protocol with the same in the published document. We could not assess the publication bias in our review because our analysis included fewer than 10 trials.

#### 2.5.7 Subgroup analyses and heterogeneity investigations

We could not perform subgroup analyses or meta-regression to explore potential heterogeneity sources due to the low number of trials in our review.

#### 2.5.8 Ethical review

Ethical approval was not required since it did not involve any primary data collection with patients or animals.

## 3 Results

### 3.1 Study selection

**Figure 1:**
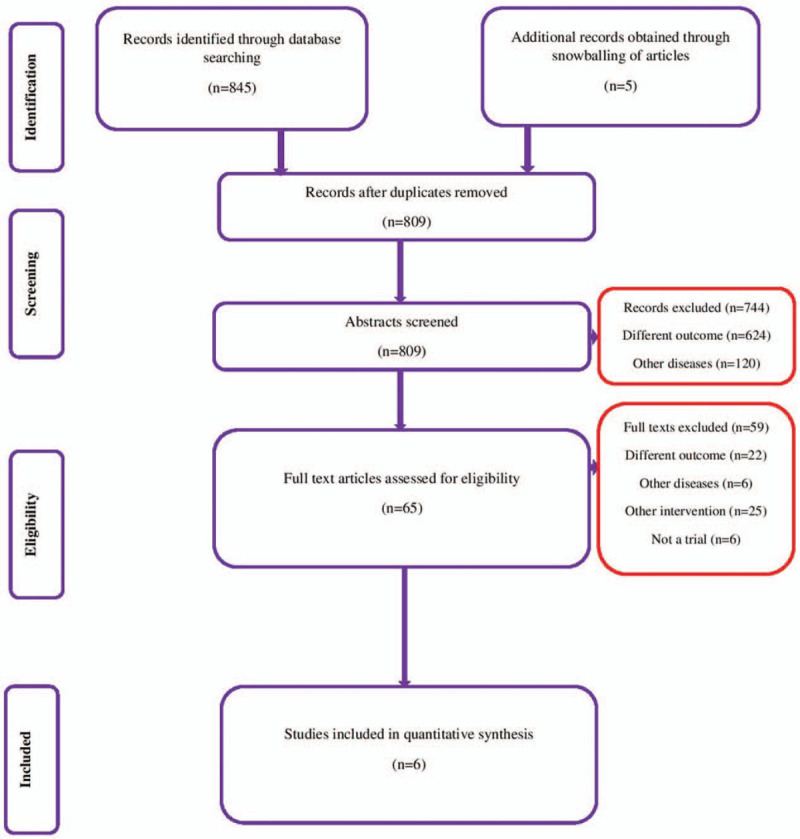
PRISMA flow chart showing the selection of studies (n = 6).

We conducted a systematic search and identified studies that directly compared the energy-restricted diet with the usual or standard GDM care diet from inception until September 2019. We obtained 845 citations and retrieved 551 trials from Medline, 121 from ScienceDirect, 114 from CENTRAL, 52 from Google Scholar, 4 from ClinicalTrials.gov, and 3 from WHO ICTRP (Fig. 1). After screening titles, abstracts, and keywords, we identified 65 relevant studies. We read their full texts and retrieved 5 studies from the bibliographies of the reviewed articles. Finally, we included 6 studies with 1300 participants satisfying the inclusion criteria.^[32–37]^

### 3.2 Characteristics of studies included

Table 1 describes the characteristics of the included trials. All the studies were RCTs and most had been conducted in high income countries like the United States, Canada, and Australia. The studies included 1300 participants (674 in the intervention arm and 626 in the control arm). The sample sizes of both study groups in each trial varied from 12 to 615 (intervention group range, from 7 to 307; control group range, from 5 to 308). Four of the 6 studies reported the modes of delivery, perinatal deaths, and macrosomia; 3 reported glycemic control data, birth weights, gestational ages at birth, and birth traumas; 2 reported large for gestational age babies, presence of hypertensive disorder of pregnancy, neonatal hypoglycemia, and shoulder dystocia.

**Table 1:** Characteristics of the included studies (n = 6).

Study number	Author and year	Country	Study Design	Sample size in intervention arm	Sample size in control arm	Intervention	Follow-up
1.	Deveer et al, 2013^[32]^	Turkey	Randomized controlled trial	50	50	The diet was tailored based on BMI by recommending caloric intakes in the range of 1800–2500 kcal/day: for women with BMIs from 20 to 25 kg/m^2^, 30 kcal/kg/d; for those with BMIs from 25 to 30 kg/m^2^, 25 kcal/kg/d; for those with BMIs >30 kg/m^2^, 15–20 kcal/kg/d.	In the intervention group, patients were followed weekly for the first month after diagnosis and every 2 weeks until delivery.
2.	Garner et al, 1997^[33]^	Canada	Randomized Controlled Trial	149	150	Women received dietary counseling and were placed on a calorie-restricted diet of 35 kcal/kg ideal body weight per day, with emphasis on spacing of meals and snacks to avoid major glucose fluctuations. Women were also taught home glucose monitoring techniques with semi-quantitative whole blood glucose reagent strips.	Women were seen bi-weekly, and biophysical profiles were performed at each visit, with ultrasonographic fetal growth, amniotic fluid volume, and cardiac size assessments.
3.	Magee et al, 1990^[34]^	United States	Randomized controlled trial	7	5	Energy-restricted diet of 1200 kcal/day diet by reducing serving sizes without changing the pattern and content of the diet in the first hospitalization week.	Daily morning double-voided urine sample for ketone and fasting plasma glucose. On the sixth day of each week: blood after overnight fast for plasma glucose, insulin, triglyceride, free fatty acids, glycerol, hydroxybutyrate. A glucose profile with 25 samples drawn over 24 h was initiated as well on the same day. On the seventh day of each wk: repeat fasting blood work (as on day 6) and a 3-h 100-g OGTT.
4.	O’ Sullivan et al, 1966^[35]^	United States	Randomized controlled trial	307	308	Low-calorie diabetic diet (30 kcal/kg ideal body weight)	Not given
5.	Rae et al, 2000^[36]^	Australia	Randomized controlled Trial	66	58	Women were placed on a diabetic diet providing between 6800 and 7600 kJ energy per day, which represented 70% of the recommended dietary intake for pregnant women (30% energy restriction).	Hyperglycemia control, blood glucose self-monitoring: before and 2 h after each meal (6 times per day) for a minimum of 2 days each wk; fetal and maternal surveillance and anticipated term birth.
6.	Yang et al, 2014^[37]^	China	Randomized controlled trial	95	55	Intensive Diabetes Management Plan—low calorie diet and exercise advice	Fortnight specialist reviews of blood glucose

BMI = body mass index.

### 3.3 Methodological quality of the trials included

Table 2 presents the assessment of the risk of bias for the included RCTs. Most studies presented unclear risks of randomization process bias (random sequence generation and allocation concealment). Only the Rae et al's, 2000^[36]^ study had performed blinding of the participants. All the studies had either high or unclear risk of bias with respect to outcome assessment blinding. Most of the studies had a low risk of bias due to incomplete outcome data except the study by Yang et al, 2014.^[37]^ Finally, excepting the study by Deveer 2013 et ak,^[32]^ all other studies had high or unclear risks of selective outcome reporting bias.

**Table 2:** Risk of bias assessment for the included studies, N = 6.

S.No	Author and year	Random sequence generation	Allocation concealment	Blinding of the participants	Blinding of outcome assessment	Incomplete outcome data	Selective reporting of outcome	Other risk of bias
1.	Deveer et al, 2013^[32]^	High risk	High risk	High risk	High risk	Low risk	Low risk	Low risk
2.	Garner et al, 1997^[33]^	Low risk	Unclear risk	High risk	Unclear risk	Low risk	Unclear risk	Unclear risk
3.	Magee et al, 1990^[34]^	Unclear risk	Unclear risk	Unclear risk	Unclear risk	Low risk	Unclear risk	Low risk
4.	O’ Sullivan et al, 1966^[35]^	Unclear risk	Unclear risk	High risk	High risk	Unclear risk	Unclear risk	Unclear risk
5.	Rae et al, 2000^[36]^	Unclear risk	Unclear risk	Low risk	Unclear risk	Low risk	High risk	High risk
6.	Yang et al, 2014^[37]^	Unclear risk	Unclear risk	High risk	Unclear risk	High risk	High risk	High risk

### 3.4 Maternal outcomes

#### 3.4.1 Glycaemic control

**Figure 2:**
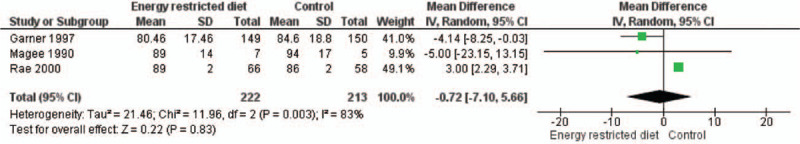
Forest plot showing the difference in glycemic control between energy-restricted diet and control groups (n = 4).

Three studies (Garner et al, 1997^[33]^; Magee et al, 1990^[34]^; Rae et al, 2000)^[36]^ reported the glycemic control statuses in both study arms. Garner et al, 1997 and Magee et al, 1990 reported that patients in the intervention arm had better glycemic control, whereas Rae et al, 2000 reported the opposite result **(**Fig. 2). The pooled mean difference was found to be −0.72 mg/dL (95% confidence interval [CI] −7.10 to 5.66 mg/dL). This indicates that patients in the intervention arm had lower mean fasting blood glucose levels by 0.72 mg/dL when compared to the levels of the patients in the control arm. But, the *P* value did not reach significance (*P* = .83), and we found no significant heterogeneity among all these studies (*I*^2^ = 83%; *P* = .003).

#### 3.4.2 Hypertensive disorder of pregnancy

**Figure 3:**
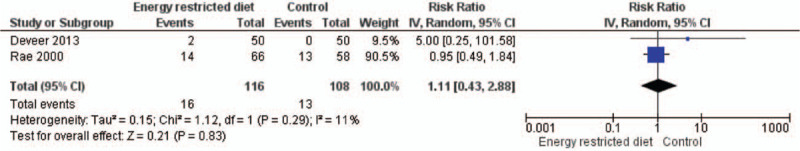
Forest plot showing the difference in hypertensive disorder of pregnancy rates between energy-restricted diet and control groups (n = 2).

Only 2 studies (Rae et al, 2000^[36]^ and Deveer et al, 2013)^[32]^ reported the hypertensive disorder of pregnancy rates in both study arms. Both showed a higher frequency of hypertensive disorders in the intervention arm than in the control arm **(**Fig. 3). The pooled risk ratio (RR) was 1.11 (95% CI: 0.43–.88). This indicates that the patients in the intervention arm had a 1.11-fold higher risk of developing a hypertensive disorder during pregnancy than the women in the control arm. But, the *P* value failed to indicate statistical significance for the association (*P* = 0.83), and we found no heterogeneity among all these studies (*I*^2^ = 11%; *P* = 029).

#### 3.4.3 Mode of delivery (Caesarean section rate)

**Figure 4:**
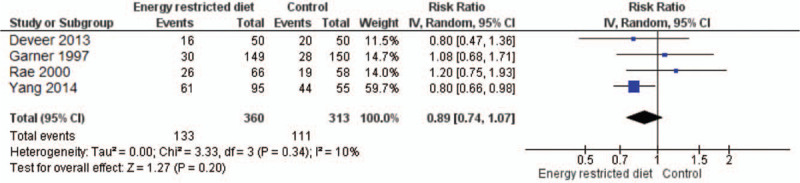
Forest plot showing the difference in caesarean section rates between energy-restricted diet and control groups (n = 4).

Four studies (Garner et al, 1997,^[33]^ Rae et al, 2000,^[36]^ Yang et al, 2014,^[37]^ Deveer 2013)^[32]^ reported the caesarean section rate in both study arms. Garner et al, 1997 and Rae et al, 2000 showed higher rates in the control arm than in the intervention arm; whereas Yang et al, 2014 and Deveer et al, 2013 reported more cesarean sections in the control group (Fig. 4). The pooled RR was 0.89 (95% CI, 0.74–1.07) favoring the intervention arm. But, the *P* value indicated a lack of significance for the association (*P* = .20), and we found no heterogeneity among all these studies (*I*^2^ = 10%, *P* = .34).

#### 3.4.4 Birth trauma

Three studies (Garner et al, 1997,^[33]^ Yang et al, 2014,^[37]^ Deveer et al, 2013)^[32]^ reported birth trauma rates in both study arms. Garner et al, 1997 and Yang et al, 2014 reported a lack of birth traumas in both study arms, only 1 woman in the control arm of the Deveer 2013 study had a birth trauma (see Figure, Supplementary Digital Content 1, http://links.lww.com/MD/F981, which illustrates the forest plot to show the difference in birth trauma between energy restricted diet and control groups). Hence, we could not pool the RR for this outcome.

#### 3.4.5 Shoulder dystocia

Two studies (Rae et al, 2000^[36]^ and Yang et al, 2014)^[37]^ reported the shoulder dystocia rates in both study arms. Yang et al, 2014 showed that the babies in both groups were all free from shoulder dystocias, only the babies of 3 women in the control arm in the Rae et al’,s 2000 study presented shoulder dystocia (see Figure, Supplementary Digital Content 2, http://links.lww.com/MD/F982, which illustrates the forest plot to show the difference in shoulder dystocia between energy restricted diet and control groups). Hence, we could not pool the RR for this outcome.

### 3.5 Fetal/neonatal outcomes

#### 3.5.1 Perinatal mortality

Four studies (Deveer et al, 2013,^[32]^ Garner et al, 1997,^[33]^ O’ Sullivan et al, 1966,^[35]^ Rae yet alt al 2000)^[36]^ reported the perinatal deaths in both study arms. All the studies except the one by O'Sullivan et al reported a lack of perinatal deaths in both study groups (see Figure, Supplementary Digital Content 3, http://links.lww.com/MD/F983, which illustrates the forest plot to show the difference in perinatal mortality between energy restricted diet and control groups). Hence, we could not pool the RR for this outcome.

#### 3.5.2 Birth weight

**Figure 5:**
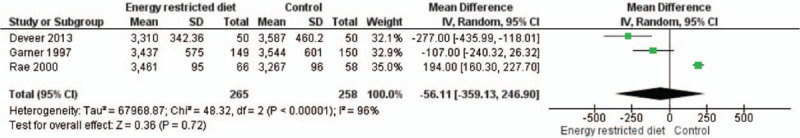
Forest plot showing the difference in birth weights between energy-restricted diet and control groups (n = 3).

Three studies (Deveer et al, 2013,^[32]^ Garner et al, 1997,^[33]^ Rae et al, 2000)^[36]^ reported the birth weights of neonates in both study arms. Garner et al, 1997 and Deveer et al, 2013 reported that patients in the intervention arm had lower mean birth weights than those in the control arm, while Rae et al, 2000 reported the opposite finding **(**Fig. 5). The pooled mean difference was −56.11 g (95% CI, −359.13 to 246.90). But, the *P* value failed to show a significant difference (*P* = .72), and we found significant heterogeneity among these trials (*I*^2^ = 96%, *P* < .001).

#### 3.5.3 Gestational age at delivery

**Figure 6:**
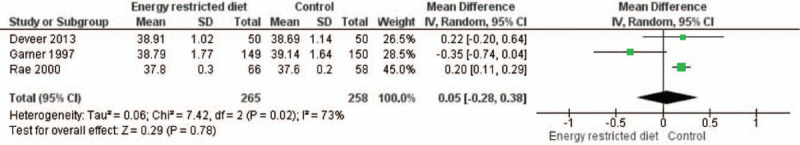
Forest plot showing the difference in gestational age at delivery between energy-restricted diet and control groups (n = 3).

Three studies (Deveer et al, 2013,^[32]^ Garner et al,1997,^[33]^ Rae et al, 2000)^[36]^ reported similar gestational ages at delivery in both study arms (Fig. 6). We found a pooled mean difference of 0.05 weeks (95% CI, −0.28 to 0.38 weeks). We found significant heterogeneity among these trials (*I*^2^ = 73%, *P* = .03).

#### 3.5.4 Large for gestational age babies

**Figure 7:**
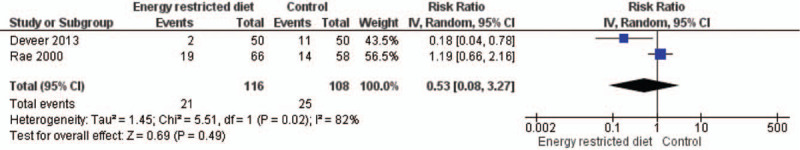
Forest plot showing the difference in large for gestational age rates between energy-restricted diet and control groups (n = 2).

Two studies (Rae et al, 2000,^[36]^ Deveer et al, 2013)^[32]^ reported the large for gestational age rates in both study arms. The pooled RR was 0.53 (95% CI, 0.08–3.27) favoring the intervention arm (Fig. 7). But, the *P* value pointed to a nonsignificant association (*P* = .49), and we found significant heterogeneity among these trials (*I*^2^ = 82%, *P* = .02).

#### 3.5.5 Macrosomia

**Figure 8:**
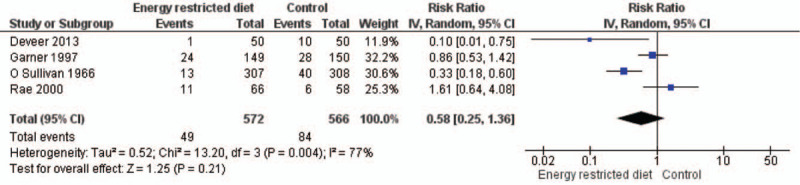
Forest plot showing the difference in macrosomia rates between energy-restricted diet and control groups (n = 4).

Four studies (O’ Sullivan et al, 1966,^[35]^ Garner et al, 1997,^[33]^ Rae et al, 2000,^[36]^ Deveer et al, 2013)^[32]^ reported the rate of macrosomia in both study arms. All the studies except the one by Rae et al, 2000 reported a lower risk of fetuses developing macrosomia in the intervention arm than in the control arm. The pooled RR was 0.58 (95% CI, 0.25–1.36) favoring the intervention arm (Fig. 8). But, the *P* value indicated a nonsignificant association (*P* = .21), and we found significant heterogeneity among all these trials (*I*^2^ = 77%, *P* = .004).

#### 3.5.6 Neonatal hypoglycemia

**Figure 9:**
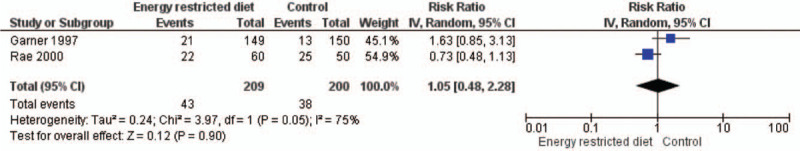
Forest plot showing the difference in neonatal hypoglycemia rates between energy-restricted diet and control groups (n = 2).

Two studies (Garner et al, 1997,^[33]^ Rae et al, 2000)^[36]^ reported the rate of neonatal hypoglycemia in both study arms. The pooled RR was 1.05 (95% CI, 0.48–2.28) favoring the control arm (Fig. 9). But, the *P* value pointed to a nonsignificant association (*P* = .90), and we found significant heterogeneity among the trials (*I*^2^ = 75%, *P* = .05).

## 4 Discussion

GDM can cause a wide range of adverse maternal outcomes, including complications during delivery, and adverse fetal/neonatal outcomes such as macrosomia, large for gestational age babies, neonatal hypoglycemia, hypocalcemia, and neonatal deaths. Not surprisingly, the most important role of primary care nurses in GDM is during timely prevention of gestational diabetes, blood glucose monitoring, interpretation of results, and maintenance of overall good glycemic control (such as recognition and treatment of hypoglycemia) that should ultimately improve overall outcomes.^[38,39]^

Different dietary interventions have been tested to reduce adverse outcomes among GDM mothers. One such intervention is an energy-restricted diet with mixed outcomes according to reports. In all, we analyzed data from six RCTs with 1300 participants residing in high and upper-middle income countries. Most trials had high or unclear risks of bias. All the maternal and neonatal outcomes, except neonatal hypoglycemia, favored the intervention arm (ie, women under energy restriction diets). However, the confidence limit crossed the null value in all the outcomes assessed and we did not find conclusive or significant evidence for any of them. This suggests that energy-restricted diets are not superior to the usual/standard GDM care diet for maternal or neonatal outcomes among women with GDM. Even though similar reviews have not been conducted on this topic, a network meta-analysis by Han et al (2017)^[40]^ comparing multiple dietary interventions including energy restriction, reported similar findings to ours.

### 4.1 Implication for clinical practice and research

Dietary counseling, often done by nurse practitioners, is considered the primary line of management for women with GDM. However, a sense of uncertainty and inconsistency persists on the effectiveness and optimal dose or duration of those dietary regimens. Our study results provide a reliable pooled estimate to solve this problem. We found that the energy-restricted diet did not produce significantly better maternal or neonatal outcomes when compared to the standard GDM care diet in the management of women with GDM. We believe that our results could be useful for a broader group of people. A wide range of GDM outcomes exist, but few trials have compared them. Thus, many results in our review were based on the data from few small trials. The impact of energy restricted diets for women with GDM on maternal and neonatal outcomes remains unclear. Studies of high quality and sufficiently powered are needed to identify significant differences in relevant maternal and neonatal clinical outcomes, and their impact on the healthcare system. These future robust RCTs or prospective studies should aim at collecting and reporting core outcomes in GDM research needed to strengthen the evidence for recommendations on how to best manage GDM patients using dietary interventions. Differing GDM diagnostic criteria and differing outcome definitions and descriptions in different studies also complicate data interpretation. Therefore, any future trials need to consider these issues.

### 4.2 Strengths and limitations

Our comprehensive literature search to gather all the relevant up-to-date publications constitute a major strength of this study. Our review synthesizes the evidence comparing maternal and neonatal outcomes between women with GDM under energy-restricted diets and those under the usual/standard GDM care diet. A network meta-analysis conducted by Han et al (2017)^[40]^ compared only three studies between these two groups of patients. We only included RCTs into our review to be able to infer causal associations between the intervention and outcomes. Since our study comprises the evaluation of RCTs performed in four different countries and four different continents (North America, Europe, Asia, Australia) and also in countries with different incomes, we believe it has good generalizability.

We are also aware of the limitations in our review. Only 6 RCTs met our inclusion criteria. This limited body of evidence assessing the effects of the intervention was insufficient to guide the clinical practice. Hence, more trials with larger sample sizes are needed. We could not assess the publication bias due to the small number of trials in our study (less than 10, the minimum requirement to perform funnel plot or Egger's tests). We did not find substantial heterogeneity for most of the outcomes in the trials. Nonetheless, we lacked an adequate number of studies to explore heterogeneity sources by performing subgroup analyses or meta-regression and we acknowledge this as a study limitation.

## 5 Conclusions

To summarize, an energy-restricted diet is not superior to the usual/standard GDM diet to improve maternal or neonatal outcomes. But, larger RCT populations are needed to attain conclusive evidence on effective, optimal doses, and optimal duration of energy-restricted dietary therapy.

## Author contributions

YF conceived and designed the study. ZZ, DF, WG and FZ collected and analyzed the data. YF was involved in the writing of the manuscript. All authors have read and approved the final manuscript.

**Conceptualization:** Yaofang Feng.

**Data curation:** Zengcai Zhao, Dayin Fu, Wen Gao, Fei Zhang.

**Formal analysis:** Zengcai Zhao, Dayin Fu, Wen Gao, Fei Zhang.

**Methodology:** Yaofang Feng.

**Validation:** Fei Zhang.

**Writing – original draft:** Yaofang Feng.

**Writing – review & editing:** Yaofang Feng.
